# On the authenticity of COVID-19 case figures

**DOI:** 10.1371/journal.pone.0243123

**Published:** 2020-12-08

**Authors:** Adrian Patrick Kennedy, Sheung Chi Phillip Yam

**Affiliations:** Department of Statistics, The Chinese University of Hong Kong, Hong Kong, Hong Kong; Institute of Tropical Medicine Antwerp, BELGIUM

## Abstract

In this article, we study the applicability of Benford’s law and Zipf’s law to national COVID-19 case figures with the aim of establishing guidelines upon which methods of fraud detection in epidemiology, based on formal statistical analysis, can be developed. Moreover, these approaches may also be used in evaluating the performance of public health surveillance systems. We provide theoretical arguments for why the empirical laws should hold in the early stages of an epidemic, along with preliminary empirical evidence in support of these claims. Based on data published by the World Health Organization and various national governments, we find empirical evidence that suggests that both Benford’s law and Zipf’s law largely hold across countries, and deviations can be readily explained. To the best of our knowledge, this paper is among the first to present a practical application of Zipf’s law to fraud detection.

## Introduction

Confirmed cases of COVID-19, the disease caused by the SARS-CoV-2 virus, were first discovered in the Chinese city of Wuhan in late December of 2019. Shortly thereafter cases began to emerge throughout Asia, Europe, and the Americas, with only a handful of countries successful in containing the disease. The unprecedented nature of the pandemic, the economic damage of which is unrivaled in recent history, has lead to calls for an international inquiry into the global response to the pandemic. Chief among concerns is the validity of case numbers reported by governments, and in particular whether falsified data lead to a failure to take the disease seriously. Operating under the Russian proverb “trust, but verify” there is a clear need for rigorous statistical techniques to detect fraud, if any, in epidemiological data. Such techniques will not only help detect fraudulent behavior, but also verify the authenticity of published case figures and hence restore trust.

Fraud detection is often predicated on the assumption that naturally occurring data sets obey certain empirical laws, and hence deviations from these laws are believed to be an indication of modified or falsified data. In the case of epidemiological data, which tracks the development of an epidemic, there are two primary ways in which empirical laws might emerge. The first is the development of the disease over time; the rate and the nature of growth in cases is of particular interest. The second is the distribution of cases over space. Given a country that is divided into sub-national regions, how should the number of cases be distributed across the country? For instance, cases that are disproportionately concentrated in a handful of regions might indicate underreporting in other areas by local authorities.

In this paper, we consider two different sources of fraud within epidemiology. The first is fraud at the national level, perpetrated by the central government, perhaps motivated by the desire to avoid travel bans and a decline in tourism, among other reasons. We propose that this might be detected using the well known Benford’s law which defines the distribution of leading significant digits in a data set, and has been observed to occur in many naturally occurring processes in the social and physical sciences. Benford’s law has long served as a litmus test in the detection of financial fraud [1–3]. More recently, researchers have considered the application of Benford’s law in identifying non-financial fraud as well, including electoral fraud [4, 5], fraudulent scientific data [6], suspicious social media activity [7], falsified law enforcement statistics [8], and fraud in international trade [9]. Therefore, Benford’s law is a natural starting point in the development of fraud detection in epidemiology. Based upon the assumption that epidemiological data obeys Benford’s law, significant deviations might then be flagged as suspicious for further investigation. At the same time, Benford’s law has also been used as the basis for evaluating the quality of data provided by public health surveillance systems. Since a reliable epidemiological surveillance system is crucial in forming appropriate policy responses during an ongoing epidemic, monitoring the performance of such systems is also an important problem. Similar to the case of detecting fraud, deviations from Benford’s law, or other empirical laws, may indicate that the surveillance system is performing inadequately. Thus, while this paper focuses on the detection of fraud in epidemiology, the theoretical and empirical results are also relevant to the problem of evaluating the performance of public health surveillance systems.

A number of empirical works have been published in recent years applying Benford’s law to epidemiology and public health surveillance. Idrovo et al. [10] applied Benford’s law to examine the performance of public health surveillance systems across countries throughout the Americas during the influenza A(H1N1) pandemic in 2009. They proposed an algorithm that used conformance to Benford’s law in conjunction with reported mortality ratios to evaluate both the quality of reported data and the sensitivity of national health surveillance systems. Their results suggested a wide range of data quality among the countries considered, and that conformance to Benford’s law was partially related to a country’s economic development. Similarly, Gómez-Camponovo et al. [11] used Benford’s law to study the dengue fever epidemiological surveillance system of Paraguay between 2009 and 2011, examining both the first and second leading digits. Interestingly, they found a much higher conformance to the second digit Benford’s law than to the first digit, highlighting the need to go beyond the most familiar version of Benford’s law. They further found that dengue fever epidemiological surveillance was best in urban areas, consistent with the earlier finding by Idrovo et al. [10] that conformance to Benford’s law was partially related to economic development. Manrique-Hernández et al. [12] applied Benford’s law to study the performance of epidemiological surveillance of Zika virus in the Americas in 2016, examining both suspected and confirmed case numbers. By using Benford’s law as a diagnostic tool, they were able to identify high performing and low performing surveillance systems, reinforcing the findings of the previous studies. More recently, Idrovo and Manrique-Hernández [13] used the first digit Benford’s law to examine China’s epidemiological surveillance system during the COVID-19 pandemic. They found that within the context of being the epicenter of the pandemic, data reported by Chinese cities, regions, and provinces indicated that China’s epidemiological surveillance system generally provided good quality data.

This paper builds on the existing works in several important ways. First, we go beyond the traditional base 10 Benford’s law by also considering smaller bases, as well as the second digit, expanding the scope of the existing empirical literature as suggested by the results given by Gómez-Camponovo et al. [11]. Second, the aforementioned studies primarily focused on countries in the Americas, whereas this paper will also examine countries in Europe and Asia that are very different culturally and developmentally. Finally, we also offer a theoretical foundation for using Benford’s law in epidemiology by arguing for its emergence using a variation of the popular susceptible-infected-susceptible compartmental model.

The second source of fraud is at the sub-national level, with falsified data provided to the central government by local authorities. We propose that Zipf’s law, another well known empirical law, might be employed to detect fraud by both local and central governments. This law emerges naturally from collections of stochastic growth processes with common mean and variance, and hence is highly suited to investigating the spread of disease among collections of similar regional populations; if we have homogeneity between regions in terms of culture, development, and demographics, then they should follow similar growth trajectories depending on the infectiousness of the virus at hand. Under the assumption that Zipf’s law holds for the geographic distribution of a disease, supported by empirical studies including this one, we propose the use of Zipf’s law as a diagnostic tool for flagging anomalous case figures. At the national level, a country with regional case figures that show significant deviations from Zipf’s law might indicate fraud by the central government. At the sub-national level, a country that largely obeys Zipf’s law with the exception of several regions may indicate fraud by the local authorities of the regions in question. The latter interpretation may find particular use by central governments in assessing the accuracy of data provided by local authorities; if the central government does not release figures by region, then it would be difficult for local authorities to falsify data since Zipf’s law concerns the relative sizes of multiple processes. Although the application of Zipf’s law to fraud detection is not as widespread as Benford’s law, we believe this is primarily due to the fact that it is often studied in fields where fraud is not a major concern. This paper therefore is among the first to present a practical application of Zipf’s law to fraud detection. Moreover, the theoretical argument that we present is based on collections of stochastic growth processes that is readily carried over to finance and econometrics, enabling the development of novel fraud detection methods in these fields as well. Finally, Zipf’s law may also be useful in evaluating the performance of public health surveillance systems at the sub-national level; namely, a few regions deviating significantly from Zipf’s law may indicate inadequate epidemiological surveillance systems in these regions relative to the rest. The application of Zipf’s law to public health surveillance is therefore a possible area of future research that builds upon this paper.

This paper is organized as follows. First, we present a theoretical argument for the emergence of Benford’s law in epidemiological data, followed by an empirical study examining the goodness of fit of COVID-19 case figures to the theoretical distribution of leading digits predicted by Benford’s law using a chi-squared test. We further provide a detailed explanation of how the observed deviations from Benford’s law are readily explained by government intervention and testing constraints. Next, we present a theoretical argument for the emergence of Zipf’s law in the geographic distribution of cases, and examine the goodness of fit to Zipf’s law using the common ordinary least squares (OLS) and log-log plot approaches, as well as the power law test proposed by Clauset et al. [14]. We discuss the limitations of fraud detection methods in epidemiology using Benford’s law and Zipf’s law, and finally summarize our findings and outline promising theoretical and empirical avenues for further research.

## Benford’s law

Benford’s law, also known as the Newcomb-Benford law, states that the leading digits of many naturally occurring processes tend to be small [15]. The law is easy to understand intuitively by considering the special case of a deterministic process that grows exponentially. If the rate of growth is constant, say some fixed percentage in each time period, then the process takes longer to surpass smaller leading digits, and does so for each order of magnitude, so that smaller leading digits are observed more frequently. Indeed, Benford’s law goes further than merely stating that the leading digits tend to be small, it also specifies the frequency distribution of leading digits, and has also been generalized to consider the *n*th leading digit.

As investigations tend to be very costly, automated methods of flagging anomalous data for investigation enables authorities to prioritize more suspicious activity. Operating under the assumption that natural or authentic data obeys Benford’s law, data is flagged as suspicious if the observed leading digit frequencies deviate significantly from those predicted by Benford’s law. Specifically, for numbers in base *b* ≥ 2, Benford’s law predicts the distribution of the first leading digits *d* ∈ {1, 2, …, *b* − 1} to be

$$\begin{array}{c} P_{b}\left(d\right)={\text{log}}_{b}(1+\frac{1}{d}) \end{array}$$ (1)

so that it is then straightforward to test the authenticity of the data using a standard goodness of fit chi-squared test by comparing the observed leading digit frequencies with those predicted by Benford’s law [16]. Moreover, if there is reason to suspect the data has been intentionally falsified to obey (1), then second or third leading digits can be tested instead. Note that the distribution of the *n*th leading digit approaches a uniform distribution exponentially fast as *n* → ∞, and hence the empirical analysis is typically limited to the first few leading digits [17]. The key assumption underlying this approach to fraud detection is that authentic data should obey (1). Absent empirical studies demonstrating this, or strong theoretical arguments in lieu of such studies, there is little evidence to support the efficacy of such an approach.

### Benford’s law and the spread of disease

An intuitive justification for the emergence of Benford’s law during the early stages of an epidemic is readily available. Let *I*(*t*) denote the number of infected individuals at time *t*, with *I*(0) = 1, and let *S*(*t*) denote the number of susceptible individuals. Suppose we consider only the early stages of an epidemic, when the upper constraint of population size is negligible. Operating under the assumption of a fixed infectiousness *θ* > 0, along with a fixed recovery rate *δ* > 0 such that *δ* < *θ*, we can describe the evolution of *I*(*t*) by

$$\begin{array}{c} I\left(t+1\right)=I\left(t\right)+\left(\theta +\varepsilon_{t+1}^{I}\right)I\left(t\right)-\left(\delta +\varepsilon_{t+1}^{R}\right)I\left(t\right) \end{array}$$ (2)

while the evolution of *S*(*t*) is analogously defined as

$$\begin{array}{c} S\left(t+1\right)=S\left(t\right)-\left(\theta +\varepsilon_{t+1}^{I}\right)I\left(t\right)+\left(\delta +\varepsilon_{t+1}^{R}\right)I\left(t\right) \end{array}$$

for *t* = 1, …, *T* − 1, so that infected individuals go on to infect, on average, *θ* healthy individuals in each time period, while infected individuals recover at a rate *δ*. The model (2) makes no assumption about whether the population is homogeneous or heterogeneous; if the population is heterogeneous, then the parameters *θ* and *δ* are simply aggregates of the infection and recovery dynamics, respectively, of the heterogeneous population. The terms $\varepsilon_{t}^{I}$ are appropriately defined independent and identically distributed (i.i.d.) random noise terms, as are $\varepsilon_{t}^{R}$. Note that the model (2) is simply the stochastic and discrete-time analogue of the popular susceptible-infected-susceptible compartmental model under the simplifying assumption that population size *M* satisfies *M* ≫ *I*(*t*), i.e., $\frac{M-I(t)}{M}\approx 1$, being in the initial stages of an epidemic (see [18] or [19] for the deterministic continuous-time version). The assumption of i.i.d. random noise is common in branching processes that are used to approximate the dynamics of the initial stages of an epidemic [18]. Writing out (2) recursively, we see that the evolution of *I*(*t*) may be expressed as

$$\begin{array}{c} I\left(t+1\right)=A_{t+1}\times A_{t}\times \cdots \times A_{1} \end{array}$$ (3)

where

$$\begin{array}{c} A_{t}≜1+\theta -\delta +\varepsilon_{t}^{I}-\varepsilon_{t}^{R} \end{array}$$

are i.i.d. random variables. As explained by Boyle [20], Benford’s law emerges naturally from “central limit-like” theorems for the mantissas of random variables under multiplicative operations and modular arithmetic. Indeed, Berger and Hill [21] show that if an i.i.d. sequence of random variables ${\left\{A_{t}\right\}}_{t=1}^{\infty }$ are not purely atomic, then $\prod_{t=1}^{T}A_{t}$ converges in distribution to Benford’s law as *T* → ∞. The form (3) then suggests Benford’s law should emerge naturally during the early stages of an epidemic.

The above explanation assumes a static infectiousness *θ*. In reality, decentralized and centralized preventative measures are likely to be implemented once the outbreak becomes severe, and hence the dynamics of disease transmission are likely to change. Moreover, even if the disease is allowed to spread uncontrollably, it will inevitably burn itself out due to the finite population size. We therefore reiterate that Benford’s law is only expected to hold in the initial stages of an epidemic, and so the time period considered must not be too large in order to ensure that $\frac{M-I(t)}{I(t)}\approx 1$, for *t* = 0, 1, …, *T*, and that major government intervention has not yet been implemented. In addition, it should be noted that asymptomatic cases might exist which tend to have a different basic reproductive number than symptomatic cases. However, the parameter *θ* is then simply a probability weighted average of the infectiousness of different groups categorized by manifested symptoms. Indeed, the main problem introduced by asymptomatic cases is the difficulty in observing them; absent a comprehensive and ongoing community testing scheme, the observed number of cases is likely to be biased in favor of symptomatic cases. Therefore, the prevalence of asymptomatic cases must be taken into consideration when making inferences.

Since we do not observe the spread of the disease directly, reported case figures will be estimates based on diagnostic tests with two primary sources of error. First, only suspected cases are tested, while the remaining cases go unobserved. Second, tests may provide false-positives and false-negatives. Logistical constraints on testing can also cause deviations from Benford’s law which are not necessarily indicative of falsified figures. If a government is constrained by a fixed number of tests per day, while the true case number increases by a rate much larger than this limit, then the cumulative confirmed cases will grow linearly, not exponentially. Berger and Hill [21] show that sums of random variables do not converge to Benford’s law, and hence constrained testing may cause erroneous conclusions. We therefore reiterate that while Benford’s law may help flag anomalous or suspicious case figures, further rigorous scientific investigation must be conducted in order to ascertain the exact cause. In particular, attention should be paid to whether deviations from Benford’s law are instead due to the public health surveillance system performing poorly, as this would likely lead to some of the aforementioned problems relating to asymptomatic cases and testing constraints.

### Methodology

In order for Benford’s law to emerge it is necessary for the process considered to cross multiple orders of magnitude. For this reason, using the traditional base 10 Benford’s law is less appropriate, since the number of magnitude changes might be insufficient over shorter time periods. Furthermore, if we employ the traditional chi-squared test, then base 10 might not provide large enough expected cell counts. Instead, it is more appropriate to focus on Benford’s law in smaller bases, such as 3, 4, and 5, though we include base 10 nonetheless. We test whether a country’s cumulative confirmed case process, in a specified base *b*, obeys (1) using the standard chi-squared approach. Specifically, letting $X_{b}^{i}\left(t\right)$ be country *i*’s confirmed case number at time *t* in base *b*, for *t* = 1, 2, …, *T*, we compare the leading digit frequencies observed up to time *T* to the expected leading digit frequencies under the assumption that Benford’s law holds. This is one of the tests used by Deleanu [8] who studies the applicability of using Benford’s law to detect false criminal enforcement statistics, and is noted in [22] as being the most common goodness of fit test used to assess the validity of Benford’s law.

Care must be taken when defining the start of the epidemic within each individual country. The start should be defined so as to best correspond to the start date of sustained community transmission. Indeed, several countries see no increase for weeks following the initial confirmed clusters, suggesting the initial outbreaks were successfully contained. Naively taking the first confirmed case as the start of the epidemic is likely to lead to erroneous results. Therefore, rather than considering the date at which the first case is confirmed, we define the start of the process $X_{b}^{i}\left(t\right)$ as the earliest date following which we observe three consecutive days of confirmed cases within the country in question. For example, although 4 confirmed cases existed in Germany as of January 28th, 2020, it is not until January 31st that we observe three consecutive days of confirmed cases. Thus, the beginning of the epidemic in Germany is defined as January 31st rather than January 28th. Likewise, although 41 confirmed cases existed in China as of January 11th, 2020, it is not until January 17th that we set the beginning of the epidemic based on the definition above.

We then consider a range of different periods *T* in order to ascertain whether conformance to Benford’s law is stable over time. Indeed, it should be noted that a weakness of the model is the ambiguity in selecting an appropriate value for *T*; the time period should simultaneously be large enough to cross several orders of magnitude, and small enough so that exponential growth has not yet been restrained due to government intervention. Therefore, we opt to consider a range of different time periods to ensure stability over time, as opposed to a single time period based on knowledge of the epidemic’s timeline. We consider *T* = 30, 40, 50, 60 since many countries included in our analysis have case numbers that are too small, i.e., did not cross many orders of magnitude, for periods shorter than *T* = 30. On the other hand, going beyond *T* = 60 is likely to lead to erroneous conclusions due to the effect of government intervention on the rate of transmission. For periods of time smaller than *T* = 30, one alternative approach would be to use the test recently developed by Moreno-Montoya [23] to test conformance to Benford’s law on small-samples, which we intend to explore in a future work that develops more formal procedures and algorithms for detecting fraud. Additionally, the development of nonparametric or semiparametric tests that utilize moving windows of various lengths to detect deviations from Benford’s law over time would be a promising area of future research.

Finally, we also consider the 2nd leading digit in our analysis; we refer the reader to [24] for the joint distribution of the first *n* leading digits, from which the 2nd digit distribution is readily derived. The data used in the following empirical analysis was published by the World Health Organization, and subsequently compiled by Humanitarian Data Exchange [25]. The accuracy of the data was confirmed by cross referencing case numbers with those published by the European Centre for Disease Prevention and Control. It contains confirmed case numbers for COVID-19 from the beginning of January, 2020, up until 20 May, 2020.

### Results

The results in Tables 1–4 present chi-squared 1st digit Benford’s law *p*-values for different combinations of *b* and *T*. In addition, Table 5 presents *p*-values for conformance to the 2nd digit Benford’s law in base 3. For the 1st digit Benford’s law analysis, it is interesting to note that as we decrease the base *b*, the number of countries that significantly deviate from Benford’s law decreases. For base 10, a total of 15 out of 60 country-time combinations are significant at the 5% level. This decreases to 13 for base 5, to 8 for base 4, and to 3 for base 3. Adherence to Benford’s law appears to depend on the base chosen, which we suspect has several causes. On the one hand, it is necessary for several orders of magnitude to be crossed to see the emergence of Benford’s law, and so base 10 might not satisfy this requirement given the limited number of days considered. Additionally, the expected cell count assumption for the chi-squared test is unlikely to be satisfied for higher digits. The effect of errors on case numbers, including errors due to testing constraints, may also be more significant for higher bases. As we increase the number of bins, in this case digits, the likelihood that errors in measurement will change the leading digit increases.

**Table 1:** Chi-squared *p*-values under base 10 case numbers.

Base 10 *p*-values				
Country	*T* = 30	*T* = 40	*T* = 50	*T* = 60
Brazil	0.108	0.396	0.149	0.503
Canada	*p* < 0.001	*p* < 0.001	*p* < 0.001	*p* < 0.001
China	0.740	*p* < 0.001	*p* < 0.001	*p* < 0.001
France	0.924	0.802	0.806	0.369
Germany	*p* < 0.001	*p* < 0.001	*p* < 0.001	0.015
India	0.928	0.870	0.880	0.995
Iran	0.904	0.776	0.378	0.085
Italy	0.914	0.950	0.946	0.227
Mexico	0.862	0.974	0.751	0.932
Romania	0.862	0.849	0.682	0.733
Russia	0.829	0.675	0.863	0.825
Spain	0.624	0.954	0.312	0.048
Sweden	0.295	0.790	0.567	0.121
United Kingdom	0.311	0.486	0.477	0.682
United States	0.010	0.033	*p* < 0.001	*p* < 0.001

**Table 2:** Chi-squared *p*-values under base 5 case numbers.

Base 5 *p*-values				
Country	*T* = 30	*T* = 40	*T* = 50	*T* = 60
Brazil	0.075	0.115	0.408	0.270
Canada	0.027	0.169	0.466	0.499
China	0.621	*p* < 0.001	0.002	0.004
France	0.753	0.617	0.782	0.171
Germany	*p* < 0.001	*p* < 0.001	*p* < 0.001	*p* < 0.001
India	0.867	0.766	0.900	0.666
Iran	0.749	0.968	0.610	0.027
Italy	0.906	0.636	0.413	0.817
Mexico	0.709	0.666	0.901	0.932
Romania	0.845	0.802	0.288	0.164
Russia	0.680	0.684	0.849	0.467
Spain	0.926	0.585	0.424	0.049
Sweden	0.532	0.777	0.426	0.570
United Kingdom	0.906	0.976	0.918	0.530
United States	0.001	0.007	0.002	0.038

**Table 3:** Chi-squared *p*-values under base 4 case numbers.

Base 4 *p*-values				
Country	*T* = 30	*T* = 40	*T* = 50	*T* = 60
Brazil	0.456	0.513	0.849	0.659
Canada	0.320	0.437	0.700	0.471
China	0.884	0.431	0.038	0.001
France	0.884	0.743	0.290	0.029
Germany	0.069	0.099	0.203	0.275
India	0.814	0.840	0.849	0.866
Iran	0.766	0.877	0.384	0.742
Italy	0.921	0.424	0.656	0.418
Mexico	0.884	0.795	0.809	0.709
Romania	0.750	0.432	0.697	0.612
Russia	0.476	0.643	0.697	0.640
Spain	0.239	0.413	0.061	0.027
Sweden	0.456	0.351	0.527	0.079
United Kingdom	0.549	0.569	0.460	0.430
United States	*p* < 0.001	*p* < 0.001	*p* < 0.001	*p* < 0.001

**Table 4:** Chi-squared *p*-values under base 3 case numbers.

Base 3 *p*-values				
Country	*T* = 30	*T* = 40	*T* = 50	*T* = 60
Brazil	0.978	0.938	0.472	0.760
Canada	0.062	0.464	0.299	0.445
China	0.978	0.218	0.029	0.003
France	0.466	0.686	0.472	0.819
Germany	0.123	0.059	0.192	0.268
India	0.978	0.938	0.650	0.566
Iran	0.466	0.803	0.456	0.760
Italy	0.999	0.664	0.987	0.293
Mexico	0.685	0.938	0.873	0.760
Romania	0.123	0.563	0.110	0.014
Russia	0.978	0.938	0.651	0.566
Spain	0.685	0.365	0.299	0.969
Sweden	0.978	0.686	0.472	0.620
United Kingdom	0.685	0.938	0.670	0.620
United States	0.123	0.564	0.894	0.819

**Table 5:** Chi-squared *p*-values for the 2nd digit Benford’s law under base 3 case numbers.

2nd Digit, base 3 *p*-values				
Country	*T* = 30	*T* = 40	*T* = 50	*T* = 60
Brazil	0.475	0.601	0.438	0.280
Canada	0.112	0.396	0.966	0.453
China	0.475	0.014	0.091	0.045
France	0.663	0.580	0.810	0.920
Germany	0.003	0.013	0.027	0.041
India	0.760	0.706	0.757	0.735
Iran	0.663	0.580	0.626	0.201
Italy	0.914	0.926	0.994	0.545
Mexico	0.994	0.914	0.948	0.988
Romania	0.934	0.717	0.963	0.545
Russia	0.794	0.395	0.581	0.717
Spain	0.501	0.418	0.208	0.983
Sweden	0.643	0.604	0.486	0.551
United Kingdom	0.845	0.924	0.693	0.780
United States	0.297	0.371	0.208	0.390

**Fig 1:**
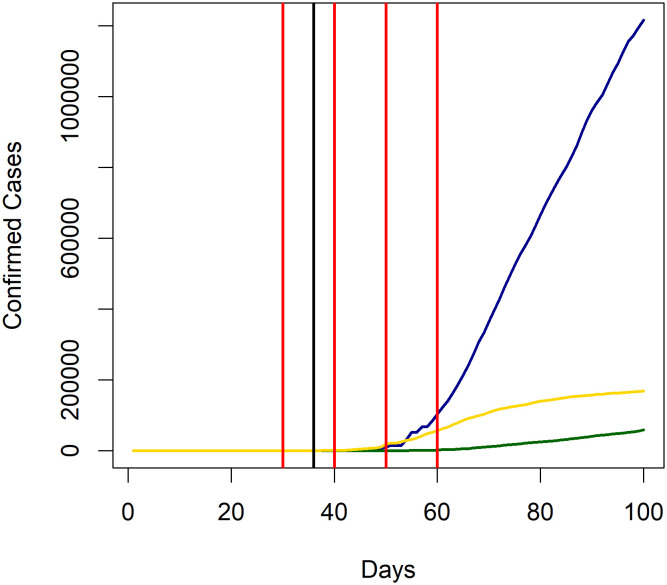
Cumulative case processes for Canada, Germany, and the United States. Cumulative case process plots for Canada (green), Germany (yellow), and the United States (blue). Days 30, 40, 50, and 60 are marked in red, while 5 March, 2020, is marked in black.

We find that Canada rejects Benford’s law in base 10 across the board, as well as base 5 for *T* = 30, at the 5% level. The cumulative case process for Canada, which shows the number of cases starting from the date defined by the 3 consecutive day rule, is given in Fig 1. From this we see that the rule of thumb we propose clearly sets the start date too early for Canada; indeed, we find that Canada has 7 cases from days 13 to 22, and 8 cases from days 23 to 27. It is not until day 36 that cases begin to rise significantly. The construction that we use to explain the emergence of Benford’s law clearly breaks down if we have large numbers of days with no new cases. Note that if we shift Canada’s start date to day 36 and consider a period of *T* = 30, we obtain a *p*-value of 0.39 for base 10. A poorly defined start date also explains why Germany rejects Benford’s law in base 10, base 5, as well as the 2nd digit Benford’s law in base 3. The cumulative case process for Germany is also presented in Fig 1, in which the same problem is evident. For instance, Germany has 16 cases from days 12 to 25 without change. If we again shift the start date to day 36 and consider a period of *T* = 30, we obtain *p*-values of 0.60 and 0.73 for bases 10 and 5, respectively, and 0.47 for the 2nd digit Benford’s law in base 3.

For the United States, we find that Benford’s law is rejected at the 5% level for times *T* = 30, 40, 50 and bases *b* = 4, 5, 10. This deviation can be explained by the failure of the Centers for Disease Control and Prevention (CDC) to adequately ramp up testing in the early days of the outbreak. Fewer than 4, 000 tests were conducted in the United States by February 28th, exacerbated by the discovery that 160, 000 tests produced throughout February were defective [26]. Moreover, the CDC relaxed testing restrictions on March 5th, after which testing increased dramatically [27]. We see in Fig 1 that following this relaxation on testing guidelines, the confirmed cases grows rapidly. The rejection of Benford’s law can therefore be explained by constraints on testing.

**Fig 2:**
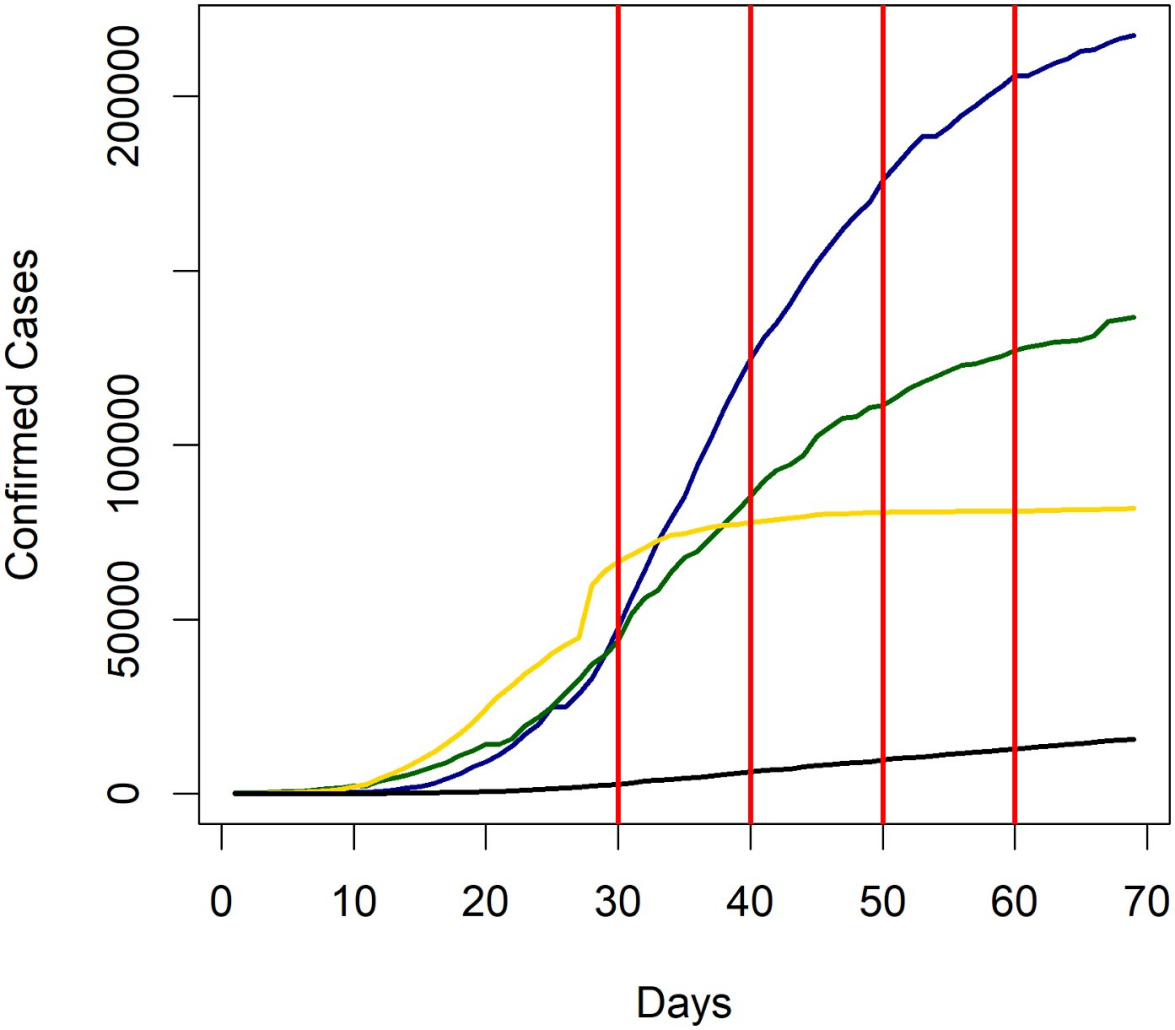
Cumulative case processes for China, France, Spain, and Romania. Cumulative case process plots for China (yellow), France (green), Spain (blue), and Romania (black) with days 30, 40, 50, and 60 marked in red.

China’s rejection of Benford’s law in all bases can be explained by a combination of being the epicenter of the outbreak, which naturally causes a backlog of undiscovered cases, difficulties in developing the initial tests, and finally strong government intervention. China’s cumulative case process, given in Fig 2, shows that between days 30 and 40 the exponential growth in cases was almost entirely halted. It is possible that the rapid growth between days 20 and 30 was the result of detecting the backlog of previously undiscovered cases; once this backlog was exhausted, strong intervention by the central government quickly flattened the growth process. According to the three day rule, China’s start date was January 17th, 2020, and just 6 days later the central government introduced lockdowns to badly affected cities in Hubei province [28]. Thus, although China’s case data displays deviations from Benford’s law in all bases considered, including both the 1st and 2nd digits, it is important to note that there are plausible explanations that do not involve fraudulent activity.

Both France and Spain only reject Benford’s law for *T* ≥ 60, and this is readily explained by government social distancing measures. The start date for France was March 2nd; subsequently on March 17th the French government initiated a lockdown [29]. This gives 45 days for the effect of the lockdown to reduce the rate of transmission by *T* = 60. Similarly, the start date for Spain was February 26th, while on March 14th a lockdown was introduced, leaving 43 days for it to affect the rate of transmission [30]. Given that Benford’s law is accepted on time periods shorter than *T* = 60, combined with the cumulative case process plots in Fig 2 which demonstrate the slowed exponential growth by this time, government intervention seems to be the most likely cause.

Finally, we find that Romania rejects Benford’s law for base 3 at *T* = 60. Referring to Romania’s cumulative case process plot in Fig 2, we find that there is no flattening of the curve as in the prior examples to indicate government intervention is the cause. Instead, the cumulative case process transitions from exponential to linear growth at around *T* = 20. As previously mentioned, constraints on testing would result in linear growth, and hence logistical constraints causing inadequate testing might explain this. However, we have been unable to find reputable sources that substantiate this explanation, and so the possibility of fraudulent activity cannot be ruled out.

In summary, we find a substantial amount of agreement between COVID-19 case figures and the leading digit frequency distribution predicted by Benford’s law. For all countries that reject Benford’s law at various bases or time periods, we find there are plausible reasons to explain the deviation, including government intervention, constrained testing, and poorly defined start dates. In fact, that Benford’s law only breaks down for some countries following government social distancing measures supports the efficacy of these measures in slowing down transmission of the disease. The results also substantiate the theoretical argument that we have presented to explain why the early stages of an epidemic should give rise to Benford’s law, and hence lay an empirical foundation for developing formal statistical fraud detection techniques in epidemiology.

## Zipf’s law

Zipf’s law is an empirical law that has been observed in many naturally occurring data sets of the social and physical sciences. Given a data set *Z*_1_ > *Z*_2_ > ⋯ > *Z*_*N*_, in which data points are labeled by their rank (*r*), Zipf’s law is said to hold if the log-log plot of ln(*Z*_*r*_) and ln(*r*) is approximately linear. Specifically, the law asserts that

$$\begin{array}{c} \text{ln}\left(r\right)=\alpha -\beta \;\text{ln}\left(Z_{r}\right)+\varepsilon_{r} \end{array}$$ (4)

for *r* = 1, 2, …, *N*, where *α*, *β* > 0, and *ε*_*r*_ is an error term. This linear relationship is also referred to as the rank-size rule; some authors, such as Gabaix [31], use Zipf’s law to refer specifically to phenomena which follow power law distributions, of which the so-called rank-size rule is a direct consequence. The law is named after the linguist George Kingsley Zipf who noticed that the frequency of a given word in a corpus of natural language is inversely proportional to its rank in the corresponding frequency table [32, 33].

Zipf’s law has been observed in many fields in the social and physical sciences, including the ranking of city sizes by population [31]. In fact, that city rankings have been demonstrated to obey, approximately, Zipf’s law is particularly surprising given the uncontrolled nature of city growth. This relationship holds in the United States throughout the 20th century [34, 35], and for most countries [36]. Indeed, a study by Moura and Ribeiro [37] found Brazilian cities also obey the law over a period of at least 50 years. The emergence of Zipf’s law in epidemiological data is of significance as it provides a practical means of flagging data for further analysis. Investigations are costly in both time and money, meaning they should only be initiated if there is good reason to suspect data of being fraudulent. Central governments compile data from hundreds or thousands of different sources, whether they be from local governments or directly from healthcare facilities, and so clearly there is a need to automate and streamline fraud detection procedures. Moreover, this applies not only for epidemiological data, but clearly for economic and financial data as well. The theoretical discussion in the next section is based on collections of stochastic growth processes with common mean and variance, much of which is readily carried over to commonly studied processes in finance and economics. Furthermore, this new approach to fraud detection is strengthened by the central government’s monopoly on information; if the government does not publish the regional breakdown of the data in question, it would be difficult for local authorities to falsify data in accordance with Zipf’s law. By monitoring the data over time, and not publishing it in detail, the central government can ascertain the expected Zipfian pattern in the data, upon which an automated fraud detection system can be developed.

### Zipf’s law and the geographic spread of disease

Since Zipf’s seminal work, multiple explanations have been proposed to explain the frequent emergence of Zipf’s law in the social sciences. We begin by presenting a brief summary of the explanation given by Gabaix [31] in the context of city population rankings, as it provides an intuitive understanding for why Zipf’s law might be expected to emerge naturally in epidemiology.

Let $S_{t}^{i}$ denote the normalized size of city *i* at time *t* among a collection of cities, the total number of which is fixed and finite. Here, the normalized city size is simply the city population divided by the entire urban population, and the initial distribution of cities is arbitrary. It is assumed that the normalized city sizes, at least over a certain range, evolve stochastically over time. Specifically, suppose that in the upper tail the evolution of city sizes is of the form

$$\begin{array}{c} S_{t+1}^{i}=\gamma_{t+1}^{i}S_{t}^{i} \end{array}$$ (5)

where $\gamma_{t+1}^{i}>0$ are i.i.d. random variables with density *f*(*γ*). Define $G_{t}\left(x\right)≜P\left(S_{t}^{i}>x\right)$ and note that

$$G_{t+1}\left(x\right)=P\left(\gamma_{t+1}^{i}S_{t}^{i}>x\right)=P\left(S_{t}^{i}>x/\gamma_{t+1}^{i}\right)=E\left[G_{t}\left(x/\gamma_{t+1}^{i}\right)\right]$$

where the third equality follows by an application of the law of total expectation. Hence the steady state process *G* = *G*_*t*_, if it exists, must satisfy

$$\begin{array}{c} G\left(x\right)=\int_{0}^{\infty }G\left(x/\gamma \right)f\left(\gamma \right)d\gamma . \end{array}$$

For a more detailed description of the above construction and its technicalities, we refer the reader to [31]. Gabaix [38] notes that possible ways of ensuring the existence of a steady state process is to add a small constant to Eq (5) to prevent cities from getting too small, or a lower bound for sizes enforced by a reflective barrier. Importantly, according to Gabaix [38] these adjustments typically do not affect the power law in the upper tail, for which only the growth rate matters. One then finds that a solution in the upper tail is given by *G*(*x*) ∝ *x*^−*β*^, with *β* > 0, provided that we also have $E[{\left(\gamma_{t+1}^{i}\right)}^{\beta }]=1$. Gabaix [31] notes that if the city sizes are normalized, then the average normalized city size is constant, and this requires $E[\gamma_{t}^{i}]=1$. Thus, it follows that Zipf’s law holds in the upper tail with *β* = 1, which is the proposed explanation for the emergence of Zipf’s law for city sizes. Gabaix [38] also points out, however, that if small cities grow much faster than larger ones, then we have *β* > 1.

The only assumption the above construction imposes on the growth dynamics is that, for a certain range of city sizes in the upper tail, the growth processes share a common mean (i.e. the average rate of population growth) and common variance. This assumption is often referred to as Gibrat’s law, and implies that the proportional rate of growth is independent of the current absolute size. The construction by Gabaix [31] shows that the steady state distribution of normalized city sizes is then

$$\begin{array}{c} P\left(S_{i}>x\right)\propto x^{-\beta } \end{array}$$ (6)

provided *x* is sufficiently large, and *β* > 0. The rank-size relationship (4) is then a consequence of this power law distribution [31]. It is easy to see that if (4) holds for normalized *Z*_*r*_, then it clearly holds for unnormalized *Z*_*r*_ by absorbing the normalizing constant into *α*.

Few empirical phenomena obey power law distributions for all values of *x*; instead, power laws typically only hold for values greater than some minimum value *x*_*min*_, which must be estimated. This can be seen in the size rankings of naturally occurring cities [39], gross domestic product [40], world cities [40], and English text frequencies [41].

Returning to the topic of this paper, consider a country comprised of a collection of regions such as states or provinces. Suppose that a common disease is simultaneously imported to each region and begins to spread locally, and *I*_*i*_(*t*) is the normalized number of infected in region *i*. That is, *I*_*i*_(*t*) is the number of infected individuals in region *i* divided by the total number of infected nationally. Considering that the disease is identical across each region, with a given level of infectiousness, and assuming that there is homogeneity between regions in terms of culture, population density, and development, it stands to reason that each region shares a common level of infectiousness *θ* > 0 and common recovery rate *δ* > 0. In this case, we again consider the form of model proposed earlier, with

$$\begin{array}{c} I_{i}\left(t+1\right)=I_{i}\left(t\right)+\left(\theta +\varepsilon_{t+1,i}^{I}\right)I_{i}\left(t\right)-\left(\delta +\varepsilon_{t+1,i}^{R}\right)I_{i}\left(t\right) \end{array}$$ (7)

for each region *i*, where $\varepsilon_{t+1,i}^{I}$ and $\varepsilon_{t+1,i}^{R}$ are again appropriately defined i.i.d. random noise terms. But clearly this can be rewritten as

$$\begin{array}{c} I_{i}\left(t+1\right)=\gamma_{t+1}^{i}I_{i}\left(t\right) \end{array}$$ (8)

where $\gamma_{t+1}^{i}>0$ are i.i.d. random variables. It follows by the reasoning of Gabaix [31] that if we have

$$\begin{array}{c} E[{\left(1+\theta -\delta +\varepsilon_{t+1,i}^{I}-\varepsilon_{t+1,i}^{R}\right)}^{\beta }]=1 \end{array}$$

then the steady state distribution of infected within each region is given by (6). Moreover, since *I*_*i*_(*t*) are assumed to be normalized, we must have

$$\begin{array}{c} E[\theta -\delta +\varepsilon_{t+1,i}^{I}-\varepsilon_{t+1,i}^{R}]=0 \end{array}$$ (9)

which suggests the emergence of Zipf’s law with *β* = 1. It is important to note that condition (9) does not mean that the number of infected isn’t increasing; since we are considering the normalized number of infected, this condition refers to the number of infected in region *i* relative to the rest of the country.

There are three obvious reasons why the above construction might break down, and which therefore must be considered when determining the cause of anomalous data. The first is that since all regions share common infectiousness and recovery parameters there is an implicit assumption of homogeneity between regions. If the regions are very different from one another in terms of culture, population density, demographics, or development, then clearly this assumption is questionable and is likely to lead to deviations from Zipf’s law. On the other hand, the model still allows for regional populations to be heterogeneous provided homogeneity between regional populations still holds. Thus, if we find that a handful of regions deviate significantly from the nationally predicted rank-size relationship, the first course of action is to check whether these regions differ meaningfully from the rest of the country. This includes whether the policy response of the local authorities differed from that of the central government, or if the response followed a very different timeline. Secondly, we have made the assumption that Gibrat’s law holds in the sense that the average rate of disease spread, along with the variance, is independent of the number of infected. However, centralized and decentralized preventative measures are likely to have an effect on the dynamics of disease spread, and the likelihood of such measures being implemented clearly increases with disease prevalence. Finally, we have assumed that the disease is imported to all regions simultaneously. In practice, it would be reasonable to expect the disease to emerge in larger transport hubs long before rural regions. The timeline of infections should therefore be examined to check whether cases in anomalous regions emerged much earlier or later than the rest of the country.

It is worth noting that one approach to falsifying data to avoid detection based on Zipf’s law is to suppress figures below the minimal value *x*_min_. After all, if Zipf’s law is observed to only hold above this value, then regions with case numbers below this value should not be flagged as suspicious for deviations from the empirical law. However, this approach has several problems. First, the value *x*_min_ is not known and must be estimated using data only available to the central authorities. Second, suppressing figures below *x*_min_ may in itself raise suspicion if *x*_min_ is sufficiently small that the region’s figures are not seen as realistic. Finally, this approach is less relevant in finance and econometrics, since fraud more often than not involves inflating figures, not understating them.

### Methodology

Clauset et al. [14] note that while the most common methods of estimating *x*_min_ are visual, i.e., constructing log-log plots and picking the point beyond which linearity appears to break down, these methods are subjective and also sensitive to noise in the tail of the distribution. As an alternative, they suggest a more objective approach which minimizes the Kolmogorov-Smirnov (KS) distance between the empirical and theoretical power law distributions, defined as

$$\begin{array}{c} K≜\underset{x\geq x_{\text{min}}}{\text{max}}|\hat{F}\left(x\right)-F\left(x\right)|, \end{array}$$ (10)

where $\hat{F}\left(x\right)$ is the empirical distribution of the data above *x*_min_, and *F*(*x*) is the power law distribution of best fit for data above *x*_min_ [42]. The proposed estimate ${\hat{x}}_{\text{min}}$ is then the value which minimizes *K*. We employ this method to obtain estimates of *x*_min_ using the R package poweRlaw.

Clauset et al. [14] also propose a test for whether data is drawn from a power law distribution above some minimal threshold *x*_min_, which is again implemented using the package poweRlaw. The test statistic used is the KS distance (10), while a semiparametric approach is used to construct a distribution for the test statistic. Having obtained point estimates $\hat{\beta }$ and ${\hat{x}}_{\text{min}}$, the idea is to generate synthetic data sets that obey the power law with scaling parameter $\hat{\beta }$ above ${\hat{x}}_{\text{min}}$, but which resemble the empirical data below ${\hat{x}}_{\text{min}}$, so that the model fitting process can be replicated in its entirety. In an ordered sample of size *n*, let *n*_tail_ denote the number of observations above ${\hat{x}}_{\text{min}}$. Then the synthetic data sets are generated by drawing a random variable from the power law distribution of best fit, which in this case uses the maximum likelihood estimator of *β*, with probability *n*_tail_/*n*, or uniformly from the data below ${\hat{x}}_{\text{min}}$ with probability 1 − *n*_tail_/*n*. For each such data set, the KS distance (10) is then calculated based on the power law distribution of best fit, and hence a distribution of test statistics is obtained. For an in-depth analysis and discussion of this test we refer the reader to [14]. A small *p*-value then suggests that there is no threshold *x*_min_ above which the data is drawn from a power law distribution.

Having obtained an estimate ${\hat{x}}_{\text{min}}$, the two most common approaches to estimating the parameter *β* in (4) are OLS and Hill’s estimator. Gabaix and Ioannides [43] note that while OLS is the most common method employed in the empirical literature, it tends to under estimate the power exponent. The alternative estimator was proposed by Hill [44], known as Hill’s estimator, and is defined as

$$\begin{array}{c} {\hat{\beta }}_{Hills}=\frac{n_{\text{tail}}-1}{\sum_{i=1}^{n_{\text{tail}}-1}(\text{ln}\left(Z_{i}\right)-\text{ln}\left(Z_{n_{\text{tail}}}\right))}. \end{array}$$

We consider in our empirical analysis the regional COVID-19 case figures as of the 11th May, 2020, for multiple countries, broken down according to region. Specifically, we consider Brazilian states [45], Canadian provinces [46], Chinese provinces [47], German states [48], Indian states and union territories [49], regions of Italy [50], states and federal territories of Malaysia [51], Mexican states [52], Romanian counties [53], Russian federal subjects [54], autonomous communities of Spain [55], Swedish counties [56], regions of the United Kingdom [57], and states and territories of the United States [58]. The data was retrieved from Statistica, which has compiled official government figures throughout the COVID-19 pandemic. We consider China with and without Hubei, since including the epicenter is likely to affect the results to some degree. Moreover, we exclude Hong Kong, Macau, and Taiwan from our analysis of China owing to the restrictions on cross-border travel and their distinct political systems.

### Results

**Fig 3:**
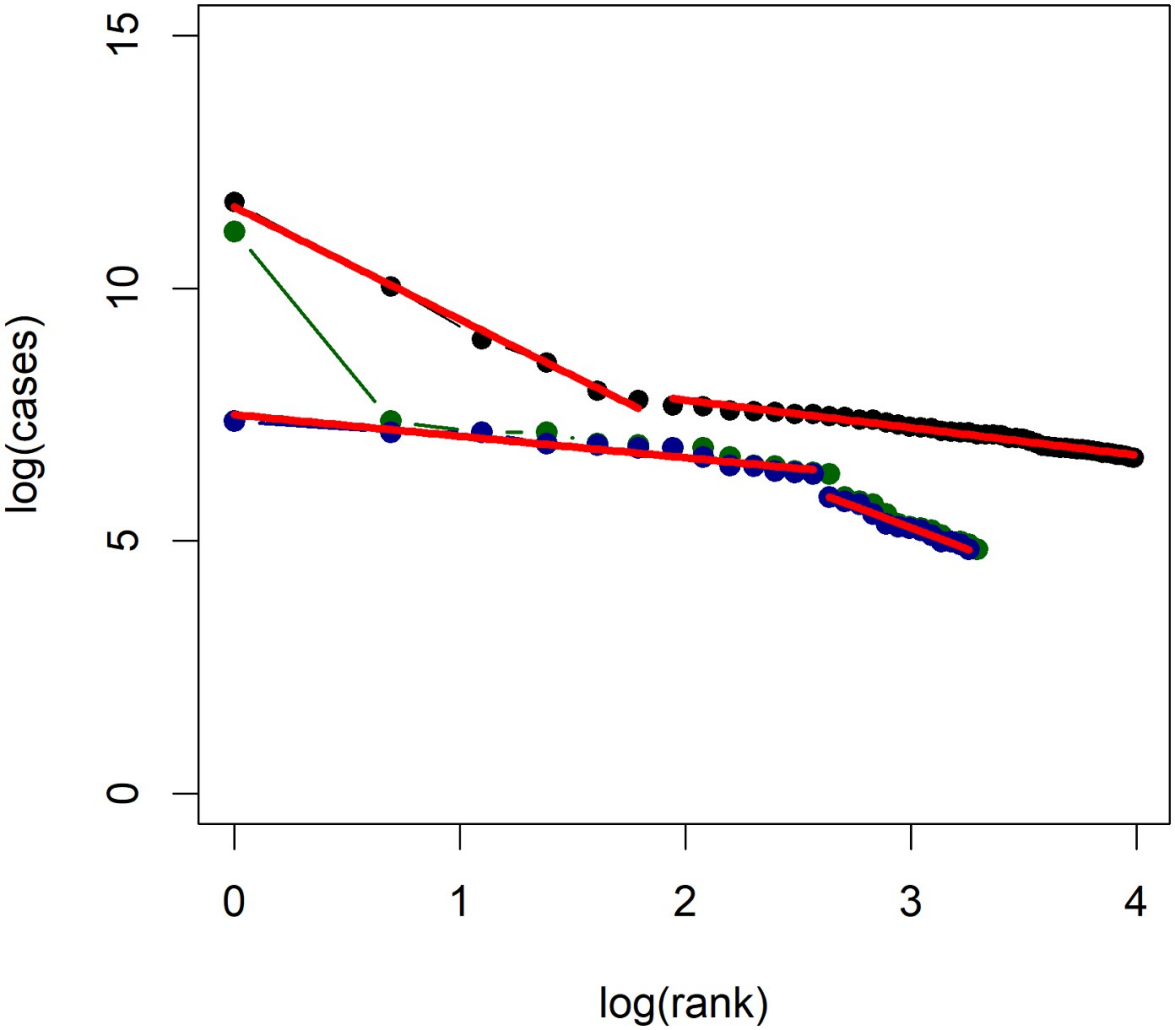
Log-log plots of confirmed COVID-19 cases in China and Russia. Log-log plots of confirmed COVID-19 cases for Russia (black), China with Hubei (green), China without Hubei (blue). Note the change in power law indicated by the change in slope.

The results of each country are summarized in Table 6, which includes the number of regions considered in the analysis after estimating *x*_min_. First of all, we find that the *R*^2^ of the model (4) is at least 0.9 for all countries except China (both with and without Hubei) and Russia; this seems to support the applicability of Zipf’s law for the regional distribution of COVID-19 cases within a nation. The low *R*^2^ also initially appears to flag case data from China and Russia as potentially unreliable, however it turns out that the low *R*^2^ for China (excluding Hubei) and Russia can be explained by a change in the power law exponent, as shown in the log-log plots of Fig 3. It seems that the procedure for estimating *x*_min_ outlined in [14] leads to taking ${\hat{x}}_{\text{min}}$ as the point at which the second power law ends, rather than the point at which the power law exponent first changes, leading to erroneous results. Such change points might be explained if they separate regions into two homogeneous subgroups, e.g., urban and rural regions, that follow similar within group growth trajectories.

**Table 6:** Regional COVID-19 cases by country.

Country	${\hat{\alpha }}_{LS}$	${\hat{\beta }}_{LS}$	*R*^2^	${\hat{\beta }}_{Hills}$	${\hat{x}}_{\text{min}}$	Number of Regions	*p*-value
Brazil	1.2957	0.8249	0.9713	0.8104	1290	22	0.75
Canada	1.7279	0.3471	0.9563	0.3448	120	9	0.92
China (Incl. Hubei)	2.7035	0.6164	0.8601	0.6997	127	27	0.03
China (Excl. Hubei)	3.4781	0.9371	0.8325	0.8098	127	26	0.01
Germany	3.5990	0.7332	0.9365	0.8463	2556	13	0.79
India	3.2996	0.7313	0.9405	0.7176	696	15	0.10
Italy	3.8109	0.7695	0.9846	0.8233	2572	15	0.99
Malaysia	2.6887	0.7676	0.9364	0.7533	89	14	0.61
Mexico	3.6844	0.9223	0.9764	1.1451	582	15	0.91
Romania	4.6407	1.3611	0.9015	1.8568	379	16	0.42
Russia	4.3869	0.9645	0.8460	1.4677	772	54	0.03
Spain	4.4858	0.9137	0.9236	1.2207	9291	8	0.73
Sweden	3.8042	0.9781	0.9209	1.4245	805	11	0.65
United Kingdom	9.9979	2.2431	0.9592	2.6948	11468	8	0.99
United States	4.8835	0.8665	0.9688	0.8212	6150	36	0.83

For Russia, the first power law applies to the regions of Moscow, Moscow Oblast, Saint Petersburg, Novgorod Oblast, Dagestan, and Murmansk Oblast. With the exception of Dagestan and Murmansk Oblast, these correspond to developed areas in the European part of Russia, which would seem to support the conclusion that the change point relates to level of development. However, Dagestan sits in the North Caucasus and Murmansk Oblast in the far north bordering Finland and Sweden, and so it is difficult to explain how they relate to the highly developed regions surrounding Moscow and Saint Petersburg. It is possible that the first cases in Russia were seeded in larger transport hubs such as Moscow and, by pure chance, also happened to be imported to Dagestan and Murmansk Oblast around the same time, so that they followed similar growth trajectories compared to regions which did not experience their first cases until much later.

Meanwhile for China (excluding Hubei) the first power law applies to Heilongjiang, Shanghai, Jilin, Inner Mongolia, Beijing, Shaanxi, Guangdong, Shangdong, Tianjin, Fujian, Shanxi, Liaoning, and Hebei. Note that these mostly comprise of coastal provinces with the exceptions of Inner Mongolia, Shaanxi, and Shanxi. Given that coastal provinces are much more highly developed, and also tend to have a higher population density, it makes sense that a change point would differentiate coastal and inland provinces. When we include Hubei into the analysis of China, the low *R*^2^ cannot be explained by a change point; the number of cases in Hubei is disproportionately large relative to the remaining provinces. However, if we assume that sustained community transmission had only just begun in the remaining provinces when COVID-19 was first discovered, the discrepancy might be explained by the significantly different growth trajectories following government intervention. The virus was allowed to spread uncontrollably in Hubei before its detection, but not in the bordering provinces, resulting in vastly different growth trajectories.

**Fig 4:**
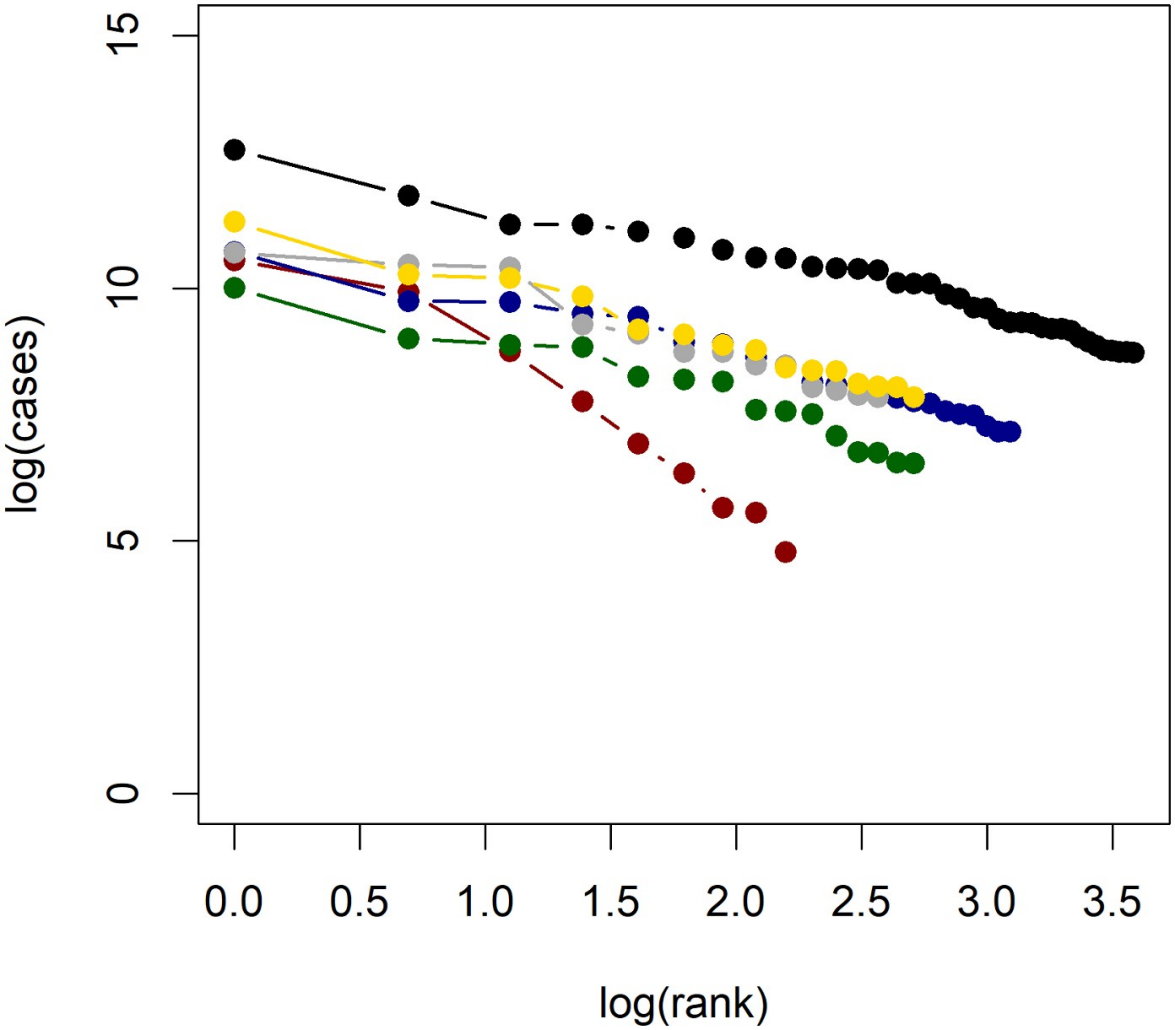
Log-log plots of confirmed COVID-19 cases in each country. Log-log plots of confirmed COVID-19 cases for the United States (black), Canada (red), Brazil (blue), Germany (grey), India (green), and Italy (yellow).

**Fig 5:**
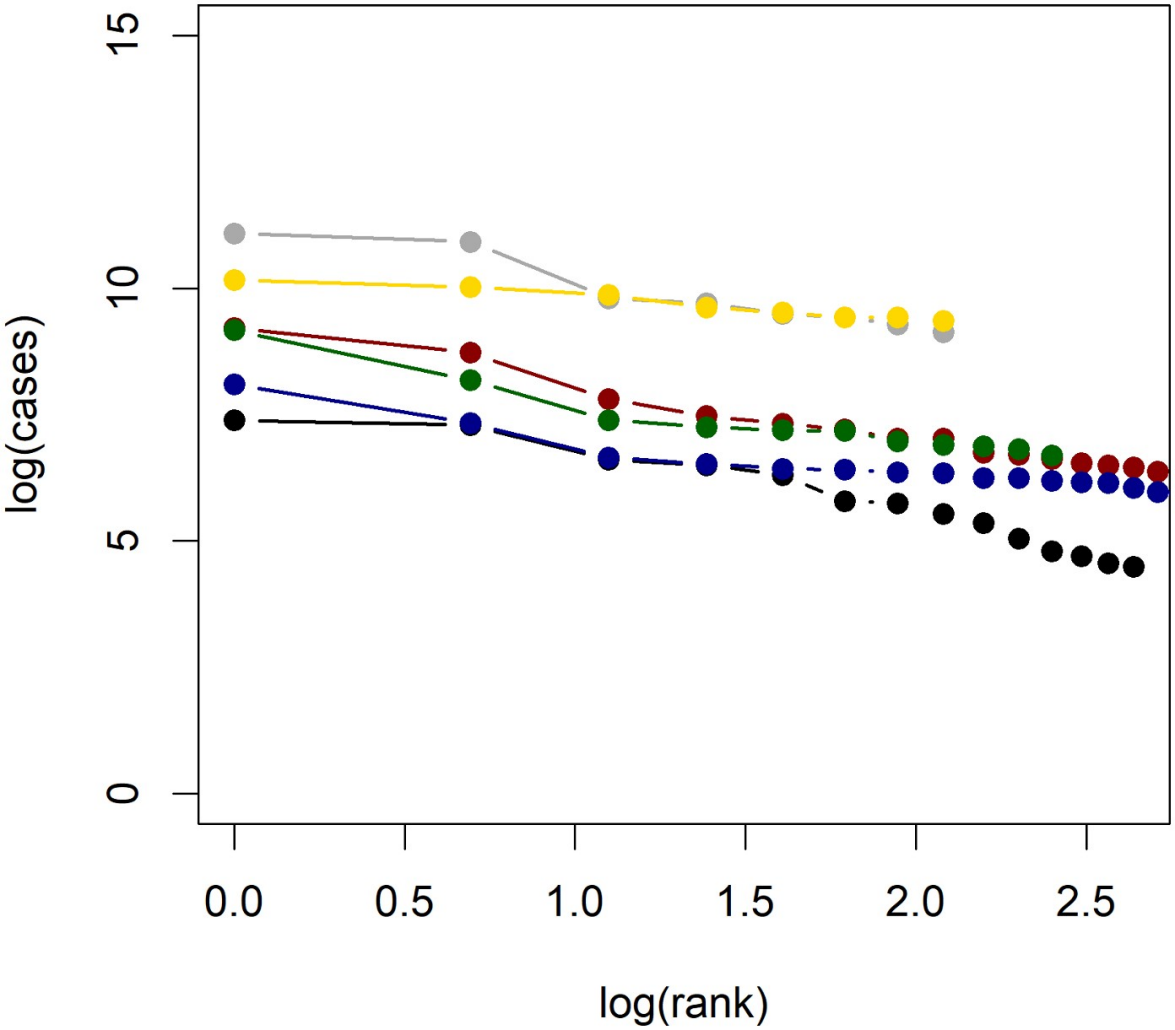
Log-log plots of confirmed COVID-19 cases in each country. Log-log plots of confirmed COVID-19 cases for Malaysia (black), Mexico (red), Romania (blue), Spain (grey), Sweden (green), and the United Kingdom (yellow).

Using the hypothesis test proposed in [14], we find that the power law hypothesis is rejected at the 5% level only for Russia and China (both with and without Hubei). That Russia and China rejects the power law hypothesis is essentially addressed by the previous discussion. In addition to the formal test outlined in [14], the applicability of Zipf’s law is often assessed visually using log-log plots. Figs 4 and 5 present log-log plots of regional COVID-19 cases for each country considered. We see that for most countries a roughly linear line is obtained, with the exceptions of China and Russia. We find that most estimates of *β* cluster around 1, supporting the theoretical argument based on the construction by Gabaix [31] which predicts a power law exponent of *β* = 1. It is interesting to note, however, that these estimates can vary quite significantly for some countries; Canada has an OLS estimate of just 0.34 while the United Kingdom has an OLS estimate of 2.24. This difference is caused by the fact that the Canadian cases are highly concentrated in the provinces of Quebec and Ontario, which together account for 84% of COVID-19 cases in Canada. Meanwhile, for the United Kingdom, the two largest regions account for only 30% of cases. Recalling that Gabaix [38] notes *β* > 1 occurs when “smaller cities grow faster,” the power law exponent estimate reflects the concentration of cases in a country. If *β* > 1, then smaller disease clusters will catch up with larger clusters, and the overall distribution of disease prevalence will tend to even out.

In summary, these results provide strong empirical evidence that disease outbreaks across a country obey Zipf’s law, albeit with a power law exponent that is not necessarily close to 1 for all countries. This provides a basis for which fraud detection in epidemiology might be based, along with a potential approach for evaluating the performance of public health surveillance systems at the sub-national level. On the sub-national level, the central government can perform statistical analysis to detect which regional governments report case numbers that deviate significantly from the observed power law. Regions that deviate significantly can then be flagged for further investigation by the relevant central authorities. On the national level, international organizations can track regional case figures of countries and flag nations which display little or no adherence to power law dynamics above any minimal threshold.

## Limitations of Benford’s law and Zipf’s law in epidemiology

There are several limitations to the approaches outlined in this paper that should be acknowledged. First, both approaches rely on a testing regime that accurately tracks the number of cases over time. If a significant number of cases are asymptomatic, then any practical testing regime is likely to be inadequate given that asymptomatic cases will generally only be detected through contact tracing and comprehensive community testing. The observed cases will therefore be biased towards symptomatic individuals, which might lead to erroneous conclusions. Hence, the approaches outlined in this paper are less appropriate for epidemics where the proportion of asymptomatic cases is large. Second, the Zipf’s law approach assumes homogeneity between regions and is therefore not suitable for countries where this assumption is violated. For instance, developing countries with large urban and rural disparities are less likely to satisfy this assumption given the impact that population density, lifestyle, and development has on the rate of transmission. Third, the models underlying each approach both assume no government intervention and a small number of cases relative to the population size. As a result, if the epidemic becomes particularly severe, applying these approaches beyond the early stages of the epidemic is likely to lead to erroneous conclusions. Finally, there are clearly some ambiguities in how the timeline of the epidemic should be defined when conducting statistical analysis based on either approach. On the one hand, the beginning of the epidemic should be set to when sustained community transmission first occurs, as opposed to simply when the first cases emerge, which might be difficult to determine with confidence. On the other hand, the end point of the analysis is also somewhat ambiguous; while we are restricted to the early stages of the epidemic, we also hope to maximize the sample size utilized. However, we note that these ambiguities could be addressed by developing nonparametric or semiparametric tests based on moving windows of varying lengths.

## Conclusion

This paper serves as a preliminary study of fraud detection in epidemiology using Benford’s law and Zipf’s law. We have presented theoretical arguments for why these empirical laws might be expected to emerge within the early stages of an epidemic, upon which statistical techniques for fraud detection may be developed. Although the theoretical constructions that we have proposed to explain the emergence of both laws rely on the same basic model, specifically a stochastic discrete-time compartmental model, each approach examines a different aspect of the data. The Benford’s law approach investigates whether the evolution of the number of cases displays anomalous behavior over time, while the Zipf’s law approach investigates whether there are any irregularities in the observed spatial distribution of cases.

The empirical results that we have presented suggest a promising degree of agreement with both Benford’s law and Zipf’s law. During the early days of an epidemic, for which the number of infected individuals is small relative to the population, the cumulative confirmed case process appears to obey Benford’s law to a large degree. Moreover, this agreement appears to hold across a variety of bases, and also to the 2nd digit. This provides a practical means of automatically flagging abnormal case numbers reported by both central and local government agencies; case figures which develop abnormally over time can then be investigated further by the relevant authorities. In addition, we find that the geographic distribution of cases across a country, above a minimal threshold, appears to obey Zipf’s law. This provides a particularly convenient method of fraud detection to central governments due to the asymmetric information possessed between central and local government authorities. Without comprehensive case numbers for other regions, local authorities would be hard pressed to falsify data that consistently obeys Zipf’s law over time.

One of the significant contributions of this paper is that we have presented a practical application of Zipf’s law to fraud detection, which has been largely neglected by the fraud detection literature. The argument that we propose to explain the emergence of Zipf’s law in epidemiology is based on collections of stochastic growth processes and so is readily carried over to commonly encountered processes in econometrics and finance, for which there is an increasing need of rigorous statistical methods of fraud detection. Unlike Benford’s law, falsifying data to adhere to Zipf’s law is difficult in the presence of asymmetric information, as the empirical law involves multiple growth processes. For fraud in epidemiology, one approach to falsifying data would be to suppress figures below the minimum value *x*_min_ above which the power law holds, however this might itself raise suspicion if case figures are seen as unrealistically low. In the case of finance or econometrics, fraud normally involves inflated figures, and so this approach is not a concern to investigators. This paper therefore presents a foundation upon which further methods of fraud detection may be developed based on the construction by Gabaix [31]. Further empirical research should study the applicability of Zipf’s law to processes in finance and econometrics for which fraud is a serious and ongoing concern.

We identify several important areas of further research that build upon this paper. First, more empirical studies should be conducted on past and future epidemics which were not met with widespread travel and social distancing measures. The actions taken by governments throughout the COVID-19 pandemic are unprecedented in scale and are likely to have an effect on the results. If the unmitigated spread of disease, based on data from prior outbreaks, shows a high degree of agreement with the empirical laws, it will help validate the theoretical arguments that we have presented. Second, operating under the assumption that the empirical laws hold, future research should develop more standardized and formal testing procedures with the aim of building a robust framework for the detection of fraudulent epidemiological data, including case studies to examine whether such methods would have detected previous cases of fraud in epidemiology. Future research could also focus on specific types of fraud, with tests tailor-made to identify that particular type of fraud. Finally, similar to the existing works based on Benford’s law, future works could explore the application of Zipf’s law to the evaluation of public health surveillance systems at the sub-national level.

## Supporting information

S1 File (ZIP)

S2 File (ZIP)
